# Multi-Drug Resistant *Escherichia coli*, Biosecurity and Anti-Microbial Use in Live Bird Markets, Abeokuta, Nigeria

**DOI:** 10.3390/antibiotics11020253

**Published:** 2022-02-16

**Authors:** Oluwawemimo Adebowale, Motunrayo Makanjuola, Noah Bankole, Mary Olasoju, Aderonke Alamu, Eniola Kperegbeyi, Oladotun Oladejo, Olubunmi Fasanmi, Olanike Adeyemo, Folorunso O. Fasina

**Affiliations:** 1 Department of Veterinary Public Health and Preventive Medicine, College of Veterinary Medicine, Federal University of Agriculture, Abeokuta 110124, Nigeria; makanjuolamotunrayo21@gmail.com (M.M.); maryvet2006@yahoo.com (M.O.); eny2be.ek@gmail.com (E.K.); oladejodotun@gmail.com (O.O.); 2 Department of Veterinary Microbiology, College of Veterinary Medicine, Federal University of Agriculture, Abeokuta 110124, Nigeria; noahbankole95@gmail.com; 3 Department of Veterinary Medicine, College of Veterinary Medicine, Federal University of Agriculture, Abeokuta 110124, Nigeria; aderonkemi493@gmail.com; 4 Department of Veterinary Laboratory Technology, Federal College of Animal Health and Production Technology, Ibadan 200262, Nigeria; bumaetal@gmail.com; 5 Department of Veterinary Public Health and Preventive Medicine, University of Ibadan, Ibadan 200284, Nigeria; olanikeadeyemo@hotmail.com; 6 ECTAD, Food and Agriculture Organization of the United Nations (FAO), Dar es Salaam 14111, Tanzania; daydupe2003@yahoo.co.uk; 7 Department of Veterinary Tropical Diseases, University of Pretoria, Pretoria 0110, South Africa

**Keywords:** antibiotics, antimicrobial stewardship program, multidrug resistant *Escherichia coli*, live bird market, Nigeria

## Abstract

Live bird markets (LBM) remain a critical link from farm to fork in the poultry value chain, which oftentimes promotes indiscriminate antimicrobial use (AMU) and resistance (AMR). In this study, we assessed biosecurity practices, AMU, and associated these with multidrug resistant (MDR) *E. coli* in LBMs in Abeokuta, Ogun State. A cross-sectional survey among live bird sellers (LBS) in eight LBMs was conducted using a semi-structured questionnaire. Also, cloacal samples (*n* = 200) were randomly collected and pooled for bacteriological detection of MDR *E. coli* in live chickens of consenting LBS. Susceptibility to 14 antimicrobials belonging to 6 different classes was determined using the disk diffusion method. Biosecurity level and AMU were generally low. LBS less than 46 years were 6.8- fold more likely to fall within the poor biosecurity level (Crudes odds ratio = 6.8; 95% CI; 1.20–38.56; *p* = 0.03) than others. An informal or primary school education increased the odds of having a poor practice of AMU by 15.1 folds (Crudes odds ratio = 15.1; 95% CI; 2.73–84.18; *p* = 0.002) than those with secondary or tertiary. The prevalence of *E. coli* and MDR *E. coli* at the LBM level were 80% and 56.3%, respectively. Extremely high resistance rates were observed for ceftazidime (96.9%) and imipenem (90.6%). The odds of MDR *E. coli* increased eight-fold in poultry kept by LBS who use AMs as prophylaxis. This current data could be useful for the development of targeted behavioral risk communication and mitigation strategies for AMR to impede the potential horizontal transfer of AMR genes to humans through animal-sourced food.

## 1. Introduction

In the Nigerian’ poultry value chain, live bird markets (LBMs) are essential to the farmers for the preferential sales and marketing of poultry and poultry products for human consumption [1]. The LBMs are typically in urban, peri-urban, and rural settings and typified by permanently placed pen-like structures in which the chickens can be temporarily housed until they are sold [2]. The LBMs bring together a variety of multi-farmed, multi-sourced, and multi-aged poultry species to meet the preferences of various customers [3]. Poultry traders and middlemen have many trade links with farms, roads, abattoirs, slaughter slabs, households, and many other locations, which have been associated with the dissemination of poultry diseases of public health importance and the persistent pool of antimicrobial multidrug-resistant bacteria pathogens [4,5]. Poor biosecurity measures in LBMs in Nigeria could create conditions that promote the spread of diseases and resistant bacterial strains [4,6]. One such common bacterial pathogen is *Escherichia coli* [7]. Many families in Nigeria depend on the consumption of poultry products such as egg and meat as protein sources, and therefore their risk of exposure to these pathogens and resistant strains potentially increase.

*Escherichia coli* are Gram-negative bacteria of the Enterobacteriales. These multi-strain organisms are widely distributed in nature, being present in soil and surface water and animal and human feces [8]. The bacteria include not only commensal strains but also pathogenic ones that cause a variety of human and animal diseases resulting directly or indirectly in more than 2 million deaths each year [9]. Pathogenic *E. coli* strains are implicated in many water and foodborne disease outbreaks worldwide, especially Shiga toxin *Escherichia coli* (STEC) and enteropathogenic *Escherichia coli* (EPEC) [10]. Since *E. coli* commonly reside in the intestines of warm-blooded animals, it is subjected to frequent encounters with antimicrobials and provides it with high selection pressure leading to a high likelihood of resistance against multiple antimicrobials consumed by its host [11]. There is the major concern of possible transmission of virulent and/or resistant *E. coli* between animals and humans through numerous pathways, such as direct contact and contact with animal excretions, through the environment, or via the food chain [8].

Antimicrobial resistance (AMR) is a major global health threat as multidrug-resistant (MDR) organisms are increasing mortality and economic burden in humans and livestock animals. Nigeria is no exception to this challenge [5]. The most common risk practice by farmers that may contribute to the emergence, selection, and dissemination of AMR microorganisms in poultry has been linked to indiscriminate antimicrobial usage (AMU) [12,13,14]. Commonly used antibiotics in poultry production in Nigeria include oxytetracycline, neomycin, enrofloxacin, doxycycline, gentamicin, colistin, streptomycin, tylosin, ciprofloxacin, nitrofurans, furazolidone, and chloramphenicol [5,12]. Several of these antimicrobials are misused and administered prophylactically either in drinking water or incorporated in poultry feeds [13,15]. Reasons for the indiscriminate use of antimicrobials in the poultry production chain were adduced as follows: (1) farmers’ efforts to reduce the burden of diseases on poultry health, (2) growth promotion in food animals to increase feed-to-muscle conversion rate and profits, (3) inaccessible and expensive veterinary services, (4) weak or non-existing antibiotic policies in the country, and (5) poor farm management or biosecurity practices [7,13,16].

AMR *E. coli* are commonly found in food producing animals such as in the gastrointestinal tract of chickens and facilities in which these chickens are housed [17,18,19]. The presence and persistence of resistance in commensal *E. coli* is a significant biomarker for the selective pressure enforced by antibiotic use and subsequent resistance predicted in other potentially pathogenic bacteria [20]. The high prevalence of MDR *E. coli* in poultry has been evaluated and reported in several countries including Nigeria [5,21,22,23,24,25]. However, only a few studies on MDR *E. coli* among chickens in LBMs in the country exist. Furthermore, to the best of our knowledge, risk factors predisposing to antimicrobial use and the consequent development of MDR *E. coli* in LBMs in Ogun State are yet to be documented. Hence, this study focused on factors associated with AMU and MDR *E. coli* in live bird markets and the potential public health implications within Abeokuta city, Ogun State, Nigeria.

## 2. Results

### 2.1. Participating Live Bird Sellers (LBS) and Live Bird Markets (LBMs) Characteristics

**Figure 1:**
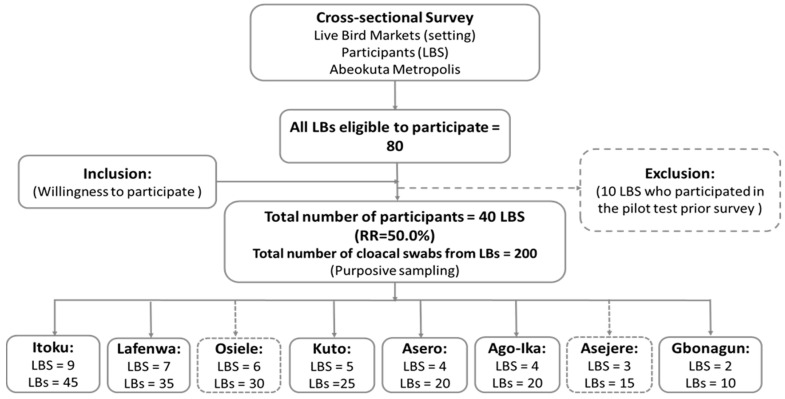
Summary study flowchart indicating the outcome of respondents’ recruitment process and sample collection.

A total of 40 LBS out of the 80 eligible participated in the study (response rate (RR) = 50%). Figure 1 represents the flow chart process for recruitment and sample collection.

All of the LBMs visited were within the Ogun Central district, one of the three senatorial districts in Ogun state. LBM capacity varied between a few hundred to several thousand chickens and the number of sellers ranged from approximately five to fifteen. The seven Abeokuta LBMs open from 7:00 am to 7:30 pm from Monday to Saturday. However, some LBS at Kuto and Lafenwa LBMs operate on Sundays.

The various types and total number of poultry present included laying hens (875, median 25), broilers (515, median 25), cockerels (380, median 20), ducks (40, median 20) and others such as pigeon and local chickens (405, median 25). Six (75.0%) of the eight LBMs visited had mixed poultry of various age groups and types. Poultry were majorly housed in metal cages 22 (55.0%). Other forms of housing were wooden (14 (35.0%)) or raffia based (4 (10.0%)). The median distance between cages was estimated at 22.5 cm (Minimum = 10, Maximum = 150). 

In all of the LBMs, poultry were fed mainly commercial feeds. Major sources of water supply described and available to respondents were boreholes (77.5%) and well (dug well) (75.0%) respectively. All LBMs had no electricity supply, no slaughtering and processing facilities despite being established mainly for selling and slaughtering live chickens.

**Figure 2:**
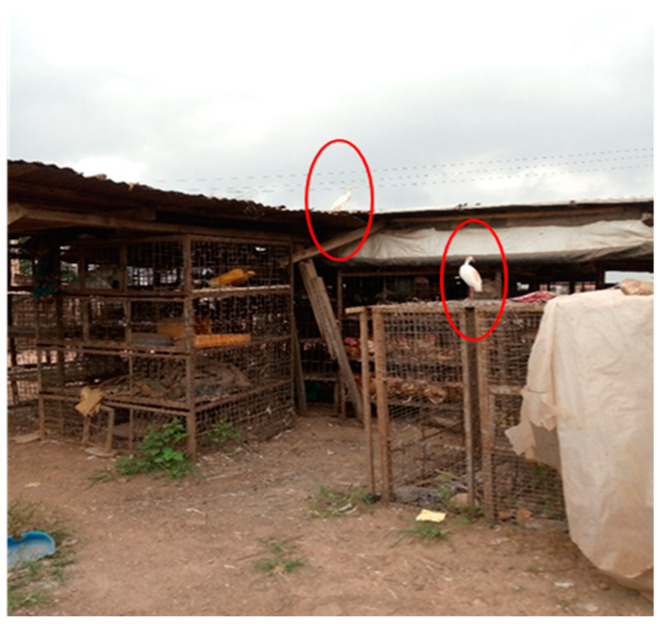
Migratory wild chickens were observed to be perching on cages close to the caged live chickens.

Live birds (LBs) were sourced from various farms and locations within and outside Abeokuta, Ogun state. These sources and distances to Abeokuta central included but were not limited to: Mile 6 (≈10 km), Ijebu (≈87 km), Yewa (≈8 km), Shagamu (≈54 km), and Remo (≈63 km). In addition, six LBMs (75.0%) get poultry supplies from contiguous states such as Oyo and Lagos, but none from neighboring countries. Seven of the LBMs (87.7%) were not fenced and were sited in residential areas (approximately 100 meters to 5/7 of the LBMs). The presence of wild chickens was also observed in the premises of 50% of the LBMs (Figure 2). Other animal types present in proximity with LBs were goats, sheep, and cattle. Dogs, cats, and rats were observed at one of the LBM during the visits (Itoku).

### 2.2. Live Bird Sellers Demographics

Out of the 40 LBS who participated in the study, 85.0% were females and 15.0% were males. The respondents’ mean age was 46 years, (SD ± 11) and the mean years as LBS was 21 (SD ± 11). The mean number of work hour per day was 10 h (SD ± 0.4), and the majority (85.0%) worked seven days a week. Most of the respondents were married (92.3%) and 45% declared that they have no formal education. None of the LBS specified their income per week (Table 1).

### 2.3. Biosecurity in the LBMs in Abeokuta, Ogun State

**Figure 3:**
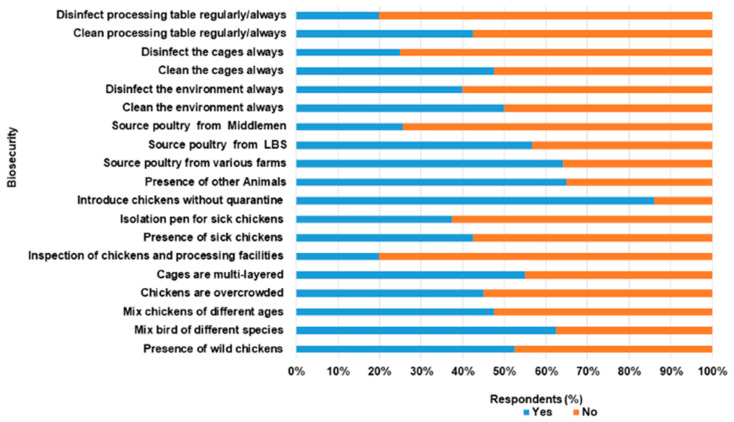
Biosecurity practices among Live bird Sellers in Abeokuta, Ogun State.

Concerning biosecurity practices within the LBMs (Figure 3), respondents confirmed that chickens were sourced from different farms. Also, chickens of different species and ages were mixed. A total of 62.5%, 55.5%, and 25.6% source poultry from different commercial farms, other LBS and middlemen, respectively. LBS (80.0%) do not carry out health inspections of poultry on arrival from the various sources and 77.5% introduced chickens into the market without quarantine. Emergency slaughter and sale, especially of sick chickens, were carried out by over 77.5% of the respondents. The presence of sick poultry was observed in the stalls of over 45.0% LBS, while over 60.0% and 55.5% do not separate sick from healthy poultry and had overcrowded poultry, respectively. Main procedures for cleaning involved the use of soap and water (22.5%), sweeping, dusting and scraping (20.0%), and use of household phenols and water (7.5%). The majority of the LBS dispose poultry waste in open dumps (70.0%). Table 2 is the distribution of responses to biosecurity questions provided by LBS who participated in the study.

The biosecurity level among LBS was poor with a mean score of 41.8% (SD 11.6%, total score 25). In this study, 70.0% of the respondents fell within the poor biosecurity level (<50.0%, range 23.3–46.7%), while others (30%) were satisfactory (>50.0%, range 60.0–66.7%). There was a weak positive correlation between the two variables, though not statistically significant (*r* = 0.23, *n* = 40, *p* = 0.15).

### 2.4. Antimicrobial Use among LBS in LBMs in Abeokuta, Ogun State

**Figure 4:**
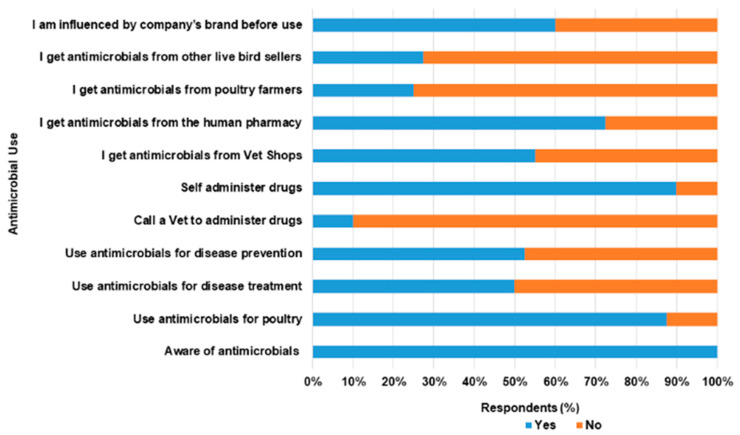
Antimicrobial use among live bird sellers in Abeokuta, Ogun State.

**Figure 5:**
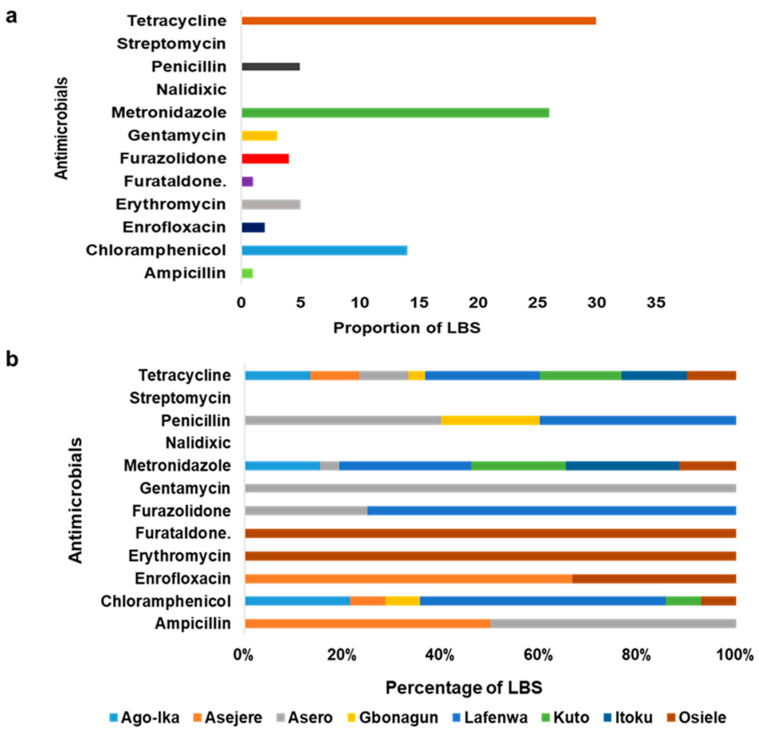
(**a**) Most commonly used AMs among LBS in Abeokuta, Ogun State. (**b**) The distribution of common antimicrobials (AMs) used across LBMs in Abeokuta, Ogun State.

Table 2 above described the responses of respondents to questions asked on AMU. In this survey, 87.5% of the respondents use antimicrobials in chickens. Out of all of the LBS, 55.0% and 20.0% use antimicrobials for therapeutics and prophylaxis purposes respectively. Figure 4 describes AMU among LBS respondents in Abeokuta. Generally, tetracycline, metronidazole and chloramphenicol were commonly used AMs by LBS in Abeokuta (Figure 5a). Figure 5b shows the distribution of AMs used among LBS across the various LBMs.

On how sick poultry were treated, only 12.5% made use of veterinary services, while the majority self-administered treatment (80.0%) and others (7.5%) patronize other live bird sellers. Common sources of purchase of antimicrobials were veterinary outlets (55.0%) and human pharmacies (27.5%). Respondents claimed that the use of AMs was influenced majorly by previous experiences (62.5%) and least by veterinarians (5.0%). Encapsulated antimicrobials targeted at humans have been used by 67.5% of the participant for treatment of diseases in poultry. A few of the human AMs used were tetracycline, metronidazole and chloramphenicol. No withdrawal period was observed by respondents (95.0%) while 100.0% were unaware of AMR. Within the period of our visits, 12.5% of the respondents still administered antimicrobials such as tetracycline, oxytetracycline, and colistin to poultry. Of these numbers, 10.0% of the respondents self- reported to have slaughtered and sold poultry to consumers within 24–48 h of treatment. 

Generally, the mean score for the practice of Antimicrobial Use (AMU) was 37.5% (SD 13.1%, Total score 30). A total of 29 respondents (72.5%) had poor practice of AMU (<50%, 12.5–45.8%), while 11 (27.5%) displayed satisfactory level (50.0–66.7%). The respondents’ preferred sources of information on Antimicrobial Stewardship Program (AMSP) in ascending order were veterinary extension services (5.0%), mass media (22.5% television and 42.5% radio) and seminars (92.5%). 

Only age was the sociodemographic variable of the LBS found to be associated with poor biosecurity level. LBS below the age of 46 years had 6.8 higher odds of having poor biosecurity level (COR = 6.8; 95% CI; 1.20–38.56; *p* = 0.03) than others. Similarly, an informal or primary school educational background increased the odds of having poor AMU by 15.1 times (COR = 15.1; 95% CI; 2.73–84.18; *p* = 0.002) than secondary or tertiary (Table 3).

### 2.5. Prevalence of E. coli and the Multidrug Resistance Profile in Live Birds (LBs) in Abeokuta city, Ogun State

The overall prevalence of *E. coli* positive samples after pooling was 80.0% (32/40; 95% CI, 64.9–89.7%). MDR was estimated in 56.3% (95% CI, 39.3–71.8%) of all putative positive *E. coli* isolates (Table 4). The other positive isolates were non-MDR (43.7%). The highest numbers of *E. coli* (28.1%, 95% CI, 15.4–45.5%) and MDR *E. coli* (33.3%, 95% CI, 16.1–56.4%) were isolated from Itoku LBM. Table 5 presents the multiple antibiotic resistance Index (MARI) estimated as 0.27 for all of the MDR isolates (ranging from 0.2 to 0.5).

**Figure 6:**
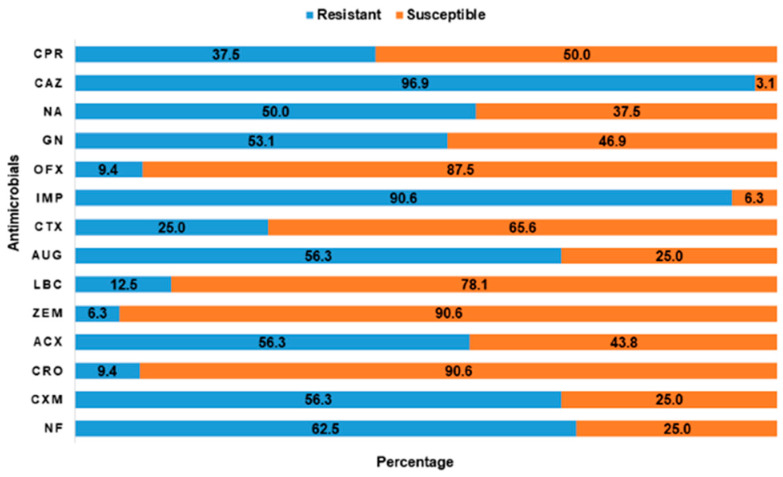
The rates of antimicrobial susceptibility and resistance pattern of putative positive *E. coli* to 14 antimicrobials tested. ACX, Ampiclox; AUG, Amoxycillin/clavulanic acid; CAZ, Ceftazidime; CPR, Ciprofloxacin; CRO, Ceftriaxone; CTX, Cefotaxime; CXM, Cefuroxime; GN, Gentamicin; IMP, Imipenem; LBC-; NA, Nalidixic acid; NF, Nitrofurantoin; OFX, Ofloxacin; ZEM, Cefexime.

Overall, extremely high rates of resistance were reported for ceftazidime (90.8%) and imipenem (90.6%). In contrast, the isolates had low rates of resistance to ceftriaxone (9.4%), ofloxacin (9.4%), and cefixime (6.3%). The overall antimicrobial resistance profile of the 32 isolates to 14 AMs is summarized in Figure 6. The distribution of antimicrobial resistance of *E. coli* isolates at the LBM level is presented in Table 6

### Prevalence of E. coli and the Multidrug Resistance Profile in Live Birds (LBs) in Abeokuta city, Ogun State

Factors Associated with MDR *Escherichia coli*

The use of AMs for prophylactic purpose was associated with MDR *E. coli* (COR = 8.05; 95% CI; 1.69–38.13; *p* = 0.01). The odds of MDR *E. coli* were eight-fold higher in poultry kept by LBS who used AMs to prevent disease compared to those that did not. In contrast, the use of AMs for treatment of LBs when sick was found to be marginally associated with MDR *E. coli* and reduced the odds of occurrence (COR = 0.17; 95% CI; 0.03–0.98; *p* = 0.067). Table 7 described the factors associated with MDR *E. coli.*

### 2.6. Feedback Meeting, LBS Perceptions towards AMU and AMR, and Challenges

A feedback meeting was organized with the live bird sellers who participated in the study. The meeting was conducted on the 18th October 2021 and a total of 12 LBS comprising of ten females and two males were in attendance. None of the LBS have had antimicrobial stewardship or poultry management training. Furthermore, the LBS shared their perceptions on antimicrobial use, resistance, and challenges experienced as live bird vendors.

When asked about their perceptions on the indiscriminate use of antimicrobials, one of the LBS responded “*We don’t use antibiotics to that extent because we purchase matured chickens, which are sold or slaughtered within a few weeks. Antimicrobial abuse is mostly from the poultry farmers because they are the ones involved in the rearing process*”. Furthermore, the respondents were unaware of the associated AMR implication in humans, but know it was critical not to slaughter chickens placed on antimicrobial treatment.

We also inquired if they observed the antimicrobial withdrawal period, one LBS responded “*Yes we do*”. Another response provided was this “*if my chickens are sick, what I do is to isolate and treat them with antibiotics. If I observe no improvement within 24 h and not to lose money, I slaughter them for my customers (consumers)*”. In Nigeria, livestock farmers use antimicrobials, but there is little compliance to withdrawal periods due to perceived economic losses. Whether LBS observe withdrawal period or not before slaughtering of chickens for human consumption will need further observational investigations.

Some of the challenges encountered within the business were also discussed. The LBS raised concerns about the lack of prompt response of veterinary services delivery and based this on the main excuse for not consulting veterinarians whenever chickens become sick. Instead, sick chickens are isolated, and treated with human capsules or turn to other sources for treatment guidance which may be unreliable, especially from poultry farmers where chickens were sourced from. The apparently healthy ones are placed on prophylactic treatment to control the spread of disease. “*We don’t seek for veterinary services when chickens are sick because of the slow response we get from them, but we call them only to fumigate the market premises*”. Lack of access to veterinary services is a common subject consistently raised as a concern among livestock producers in the country, and one of the perceived reasons for antimicrobial misuse [1,26,27,28]. Although AMU in animals for prophylaxis and metaphylaxis may have been substantially reduced in high-income countries, it is still a challenge in developing countries.

Lastly, the respondents decried the presence of unregistered live bird sellers bringing various chickens from unknown locations to the open markets especially during the festive periods consequently sabotaging the profits of the registered ones. “*We want the government to stop unregistered live bird sellers from sabotaging our efforts and bringing in diseases to our chickens. Only registered LBS should be permitted to sell chickens*”. LBS further suggested that for progress in the live bird market in the state, the government needs to fund the business, upgrade or modernize the markets to internationally acceptable standards with the provision of guidelines for good biosecurity, adequate processing facilities, basic amenities such as electricity, potable running water, drainages, good and accessible roads, which are all currently lacking. They also requested veterinary services should be prompt, accessible and affordable.

## 3. Discussion

Live bird markets (LBMs) are critical points for surveillance of indiscriminate use of AMs and MDR pathogens of public health importance. They (LBMs) serve as sources of AMR propagation to consumers of LBs, especially since many families in Nigeria depend on poultry products, which are purchased freshly slaughtered or live as a source of protein [29,30]. It is thus crucial to consider LBMs as surveillance points for the assessment of biosecurity, AMU, AMR. The knowledge derived therefore should assist in developing empirical and specific mitigation measures and effective antimicrobial stewardship program in the poultry industry, including the development of management guidelines for livestock farmers in Nigeria.

The biosecurity and practices of AMU among the LBS surveyed were generally poor. This data supported a preliminary study [1], which reported that majority of LBS had poor biosecurity and AMU in Abeokuta, Ogun State. As with the previous study [1], the presence of wild migratory birds around the live chickens bridges biosecurity and poses a risk to human and animal health and food safety. Wild migratory birds may transmit antimicrobial resistant strains to waterways, food animals, and the environment via possible interaction and fecal contamination [31]. When AMR bacteria colonize wild animals, and especially migratory birds, they become a reservoir and vectors that disperse these bacteria and AMR genes to new localities [31]. Wild migratory birds have been implicated in the introduction, maintenance, and global dissemination of different pathogens of transboundary animal diseases and zoonoses such as Highly Pathogenic Avian Influenza (HPAI), Newcastle disease, and AMR-bacteria such as MDR *E. coli* [32,33,34,35,36,37,38].

Operable biosecurity standards within the livestock industry in Nigeria is still a constraint against the effective control of animal diseases. Poor biosecurity measures and animal health management may result in farmers’ overdependence on AMs as a disease prevention strategy [13,30]. It is suggested based on this study that there is the need for stakeholders in the food animal industry to have a framework that will identify, prioritize, develop, implement, and enforce guidelines for the best practices for managing live animal farms and markets. Such guidelines or vademecum of the best practices for livestock owners may promote the responsible use of AMs in the study area. The only factor found to contribute to poor biosecurity was the age of live bird sellers, which suggests that there may be the need to intensify targeted educational intervention on the best farm practices and biosecurity especially among LBS below the middle-aged category (this constituted the majority of LBS) in the studied area. Improving literacy on biosecurity and developing and enforcing poultry management standards in LBMs may be critical in reducing indiscriminate use of AMU. However, more scientific data may be needed to promote the best farm practices and the adoption of a set of attitudes and behaviors along the poultry value chain to reduce risks in all activities of the poultry production and marketing systems [39].

Self-medication of sick poultry with common antimicrobials such as tetracycline, chloramphenicol metronidazole, and furazolidone by LBS was reported. It should be noted that such antimicrobials may be purchased from both regular and veterinary pharmacies. In addition, anecdotal evidence revealed that humans may sometimes not complete the dosages of their antimicrobial treatment and the unfinished human antimicrobials may be used to treat animals. These individuals often rely on previous experience without resulting to a proper diagnosis in a veterinary laboratory, and the accompanying antimicrobial sensitivity testing, with consequences on engendering and promoting resistant pathogens. Live bird vendors patronizing regular pharmacy stores and using antibiotics intended for human use in poultry may have detrimental effect on the effectiveness of antibiotics that are important for human medicine and the risk of selecting for drug-resistant bacteria that may spread to poultry consumers. Furthermore, previous reports have raised concerns about the administrations of AMs without veterinarian’s prescriptions and livestock farmers over reliance on previous experiences to treat animals. This is a common practice by livestock owners in Nigeria, who often inadvertently understudy and repeat past treatment administered by the veterinarians/para-veterinarians [13,27,40,41,42]. In addition, the use of banned drugs with known genotoxic and carcinogenic effects on humans for food animal production in the country is worrisome and there is the need for this to be reviewed by the National Agency for Food and Drug Administration and Control (NAFDAC) and the other veterinary governing bodies in Nigeria. Based on these findings, it is crucial to develop and enforce strictly national AMU and AMR policies through one health intervention to enhance prudent use of antimicrobials in both human and animal medicine and AMR containment. Furthermore, exclusive collaboration among the Federal Ministry of Health, Ministry of Agriculture and Rural Development, and Ministry of Environment is needed to promote AMR policy and plans towards improving awareness and understanding of AMR through effective risk communication, education and training of stakeholders and the populace.

On antimicrobial resistance (AMR), the World Health Organization recognizes this as a major international health threat [43]. A global projection predicts that the increase of deaths linked to AMR may rise to 10 million deaths in 2050, while 100 trillion USD could be lost [44]. Though the poultry sector is an important source of nutrition, food security, and financial income, it is still faced with the challenges of poor disease control and biosecurity, indiscriminate use of AMs and AMR [45]. The issues of misuse and overuse of AMs and AMR are especially significant in developing countries such as Nigeria, where multiple antimicrobials are not only used to treat infections but prophylactically and as growth promoters [45]. To support this, the MARI estimated in this study indicated MDR *E coli* isolates originated from high-risk source of contamination where various antimicrobials were used [46,47].

The importance of infections due to multidrug-resistant (MDR) *E. coli* has been increasingly recognized in recent years and they are associated with increased morbidity and mortality [48]. *E. coli* also represents a major reservoir of resistance genes that may be responsible for treatment failures in both human and veterinary medicine [8]. According to the World Health Organization [49], resistant Gram-negative bacteria (especially *E. coli*) have become a major and rapidly increasing problem globally. Presently, there are no new classes of antimicrobials in the pipeline, and so it is unlikely that any new classes of effective antimicrobials will be available for 10 years or more to treat infections caused by resistant Gram-negative bacteria [45]. MDR *E. coli* in food animals in Nigeria is becoming prevalent [50,51], and several papers have documented MDR *E. coli* in poultry and poultry products in the country [29,52,53,54]. The potential risk of spread of MDR to humans through poultry consumption has been established in a case control study conducted in Canada [55]. Another study from Ghana demonstrated that four human isolates and broiler isolates were closely related suggesting a possibility of spread of resistance between the two populations [56]. In Nigeria, a study published in 2019 demonstrated that occupational exposure to poultry on farms and the live bird market environment for over 10 years was associated with acquiring MDR *E. coli* in humans [5]. On a global scale, *E. coli* is the most important human pathogen and causes substantially many more infections than *Salmonella* and *Campylobacter* combined [49]. Thus, the importance of resistance in *E. coli*, typically considered a benign commensal, should not be underestimated [49].

Lastly, the resistant pattern of *E. coli* from this study was observed to be consistent with those reported in poultry farms in Abeokuta by a previous author [52]. Nearly all of the isolates were resistant to ceftazidime and imipenem and suggested the presence of potential extended spectrum beta-lactamase (ESBL) and carbapenemases producing *E. coli*. There may be a need for further molecular studies to establish this hypothesis. Extremely high rates of resistance to ceftazidime (third generation cephalosporin) and imipenem (carbapenem) by *E. coli* isolates in this study is worrying since this is an emerging concern in veterinary medicine as these drugs are listed by the WHO as critically important for human medicine [57,58]. Antimicrobials such as extended-spectrum cephalosporins (ESC) and carbapenems have been categorized by the World Health Organization (WHO) as a last resort and critically important antimicrobials (CIAs), with limited alternatives in the cases of resistance development. The WHO has also identified these pathogens as crucial for further research and development of new antibiotics, for instance, carbapenem-resistant enterobacteriales [59]. Similarly, the Centre for Disease Control (CDC) had recommended that care facilities should establish a protocol, in conjunction with Clinical and Laboratory Standard Institute (CLSI) guidelines, to detect resistance and carbapenemase production in Enterobacteriales particularly *Klebsiella* spp and *E. coli* and immediately alert epidemiology and infection staff members if identified [60,61]. Carbapenems, such as imipenem and meropenem, are antibiotics with a broad spectrum of activity compared to other β-lactam classes and reserved for known or suspected severe multidrug resistant bacterial infections. Although carbapenem resistance is mediated by a variety of mechanisms and its scope and transmission in livestock and to humans and in either direction is rarely performed or reported [19,62,63].

## 4. Materials and Methods

### 4.1. Study Area and Study Population

**Figure 7:**
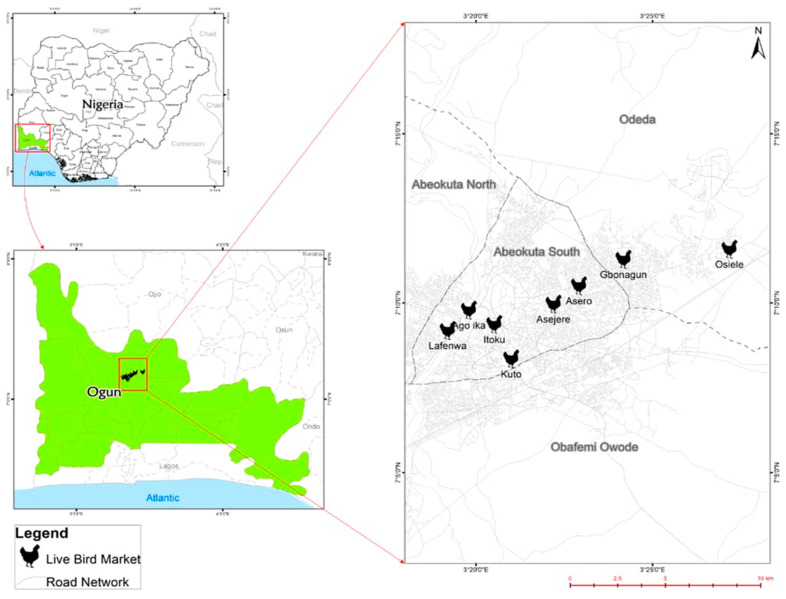
Spatial distribution of LBMs surveyed in Abeokuta, Ogun State.

The study was conducted in Abeokuta, capital of Ogun State, in the South-West region of Nigeria. Eight LBMs within Abeokuta were included: Gbonagun, Kuto, Lafenwa, Itoku, Ago-ika, Asero, Osiele, and Asejere (Figure 7).

### 4.2. Study Design and Sample Size Estimation

A cross-sectional study of the eight LBMs within Abeokuta city was conducted from March till August 2021. The target populations were the entire LBMs and LBS in the Abeokuta metropolis. The live bird markets were purposively recruited from the list of LBMs provided by the Department of Livestock, Ministry of Agriculture, Ogun State, Nigeria. All LBMs from the list of registered markets (five) within Abeokuta metropolis were recruited. Three unregistered LBMs were also included to ensure all LBMs within Abeokuta metropolis were captured.

The adjusted sample size of LBS was estimated using WinEpi. The following parameters of 5% accepted level of precision, expected prevalence of 50%, known population of LBS (100) at 95% CI were imputed to give an estimated size of 80 LBS. To detect pathogen (MDR *E. coli*) at an expected minimum prevalence of 65% at 5% precision and 95% confidence interval (assuming a population ranging between 50–100 chickens per LBS and adopting simple random sampling), a minimum of five chickens were randomly sampled from each participating LBS.

### 4.3. The Recruitment of Live Bird Markets (LBM) and Live Bird Sellers (LBS)

Pre-study visits were made to the Poultry Farmers Association, Ogun State and the live bird market (LBM) coordinators. Signed consent forms were obtained from the two parties. From each participating LBS, informed consent was obtained verbally and witnessed by the LBM coordinator. All respondents in the study were given detailed information about the aims and benefits of the study. Participation in the study was voluntary, and as many as were willing to participate were recruited. Personal identifiers were not collected and each participant’s information was treated with confidentiality. Every participant was notified of his/her right to discontinue participation at any stage of the study according to the World Medical Association Declaration of Helsinki, 2001.

### 4.4. Questionnaire Design and Data Collection

The questionnaire used for this survey is a modified version from studies conducted by Adebowale et al., 2016 and Aworh et al., 2019 [5,12]. Permission to modify survey tool was requested and granted by Dr. (Mrs) Mabel Aworh-Ajumobi, an assistant Director/Epidemiologist, Veterinary Drugs Monitoring/Animal Welfare Branch, Quality Assurance and Standards Division, Department of Veterinary and Pests Control Services, Federal Ministry of Agriculture and Rural Development, Abuja, Nigeria. The questionnaire used in this survey was divided into four sections. The information gathered included general data on the LBM, sample flock and biosecurity, live bird seller’s demographics, antimicrobial use and resistance, and preferred information channel on antimicrobial stewardship/use and biosecurity. Supplementary File S1 provides further details on the questionnaire. The standardized semi-structured questionnaire was pretested among 10 LBS (who were excluded from the study) and later administered to consenting LBS.

### 4.5. Sample Collection

In total, 200 cloacal samples were obtained from live chickens of consenting LBS (40), which were later pooled. Briefly, five chickens were randomly selected from each cage and sterile cloacal swab samples were taken, packed individually, and transported on ice packs to the laboratory of the Department of Veterinary Microbiology, College of Veterinary Medicine, Federal University of Agriculture Abeokuta, Ogun State within 2–4 h after sampling. All microbiological investigations were conducted in this laboratory. 

### 4.6. Laboratory Isolation and Identification

#### 4.6.1. Non-selective and Selective Enrichment of Samples

Swabs were broken into tubes containing 2 mL Buffered Peptone Water (BPW, Oxoid, Basingstoke, United Kingdom), vortexed for 2 min, and later incubated for 24 h at 37 °C. A loop-full of the bacteria suspension was streaked on MacConkey Agar (MA, Oxoid, Basingstoke, United Kingdom) plates and incubated at 37 °C for 24 h. Putative positive *E. coli* colonies usually pink to red were identified, selected randomly, then sub-cultured on Nutrient Agar (NA, Oxoid, Basingstoke, United Kingdom) plates and incubated at 37 °C for 24 h under aerobic conditions for the isolation of pure isolates. 

#### 4.6.2. Biochemical Identification of *E. coli* Isolates

The putative positive *E. coli* colonies were streaked on nutrient agar plates for biochemical confirmation. Conventional biochemical tests such as Gram staining, catalase, oxidase, indole, citrate, motility, triple sugar iron were performed for further phenotypic screening of selected pure *E. coli* colonies as described previously [64].

#### 4.6.3. Antimicrobial Susceptibility Profiling

The antibiotic susceptibility patterns of *E. coli* isolates were tested using the Kirby Bauer disk diffusion method and according to the Clinical and Laboratory Standards Institute guidelines [65]. All putative positive isolates were tested using a panel of 14 antimicrobials (Celtech Diagnostics, Belgium) of 6 different classes commonly used in the treatments of human and animal bacterial infections (Table 8). Briefly, one colony of the test isolates from overnight cultures grown on Eosin-Methylene Blue (EMB, Oxoid, Basingstoke, United Kingdom) agar plates were sub cultured onto NA plates for 24 h at 37 °C. Subsequently, Colonies *E. coli* were emulsified into sterile saline to achieve turbidity equivalent to 0.5 McFarland standard, which is equivalent to 10^8^ CFU/mL. Suspensions were spread using sterile cotton swabs onto Mueller Hinton (MH) agar plates (Oxoid, Basingstoke, United Kingdom), allowed for 5 min to dry, and the antibiotic discs (Table 8) were aseptically placed onto the surface of the MH agar using sterile forceps, and incubated at 37 °C for 24 h.

The zones of inhibition (diameters) were measured, recorded, and the values were interpreted using standard recommendations of the Clinical and Laboratory Standards Institute. The concentrations and cut-off limits for antimicrobials used for susceptibility testing and outcomes for *E. coli* isolates are presented in Supplementary Tables S1 and S2. For the quality control (QC), *E. coli* ATCC 10536 was used.

A strain was considered “resistant” if resistance was observed to at least one antimicrobial agent tested. Also, an isolate was defined “multi-drug resistant” (MDR) if it displayed resistance to three or more classes of antimicrobials [64].

#### 4.6.4. Rates of Antimicrobial Resistance (AMR)

To calculate the rate of resistant isolates per 100 for each antimicrobial, we performed the following calculation

(1) $$\%\;\mathrm{rate}=\frac{\mathrm{Number}\;\mathrm{of}\;\mathrm{resistant}\;\mathrm{isolates}*100}{\mathrm{Number}\;\mathrm{of}\;\mathrm{tested}\;\mathrm{isolates}}\;$$

According to Papadopoulos et al (2021), resistance % rates were categorized as extremely high (% rate >70%), very high (% rate: >50 to 70), high (% rate >20 to 50), moderate (% rate >10 to 20), low (% rate >1 to 10), very low (% rate 0.1 to 1), and rare (% rate <0.1) [66].

#### 4.6.5. Multiple Antimicrobial Resistance Indices (MARI)

The multidrug resistance level was quantified using the multiple antibiotic resistance indices (MARI) according to the formula described by the previous author [67].

(2) MARI=a/b

Where *a* = total number of antibiotics to which an isolate shows resistance and *b* = total number of antibiotics to which the isolate was exposed.

### 4.7. Data Analyses

Data analyses were performed for all variables and presented in frequencies and proportions/percentages using Microsoft Excel^®^ (2013), GraphPad Prism 9.20, and SPSS version 23.0. Continuous variables such as number of poultry types, distance between cages, age of LBS, number of work hours, AMU, and biosecurity scores were tested for normality using the Shapiro-Wilk test (>0.05), which informed our use of (Mean ± SD or Median). To evaluate the biosecurity and AMU level of respondents, a numeric pattern of scoring was used by giving a score of “1” for the “correct answer” and “0” for an “incorrect” or “I don’t know”. Respondents’ biosecurity and AMU total scores were arrived at by summing correct responses, generating a maximum possible score of 25 and 30 respectively.

The biosecurity and AMU scores were converted to percentages. Thereafter, the overall biosecurity and AMU scores among study respondents were re-grouped into two levels using <50% (Poor) and ≥50% (Satisfactory) as cut-off points [68]. The normal Q-Q plot, Shapiro-Wilk test, and simple scatter plot were performed to test the normality and linearity of the distributions of biosecurity and AMU scores and Pearson product-moment correlation coefficient was performed to measure the strength and direction of the linear relationship. The relationship between biosecurity and AMU was investigated using a Pearson product-moment correlation coefficient. Preliminary analyses were performed to ensure no violation of assumptions of normality and linearity.

Associations between the socio-demographics of LBS, biosecurity and AMU were determined using bivariate analysis such as Pearson’s Chi-Square or Fischer’s exact test (where cells have expected count less than 5). Biosecurity and AMU variables associated with MDR *E. coli* were further investigated. Outcomes were considered significant at *p* ≤ 0.05. Risk estimates at 95% CI were reported. 

## 5. Conclusions

Reducing antimicrobial consumption and misuse in animals and humans is critical to decreasing the threat of AMR in Nigeria. In this study, AMU and biosecurity practices among LBS in Abeokuta, Ogun state were generally poor. The presence of MDR *E. coli* in LBMs in the study area suggests that the bacterial organism from poultry source can contribute significantly to the horizontal spread of multi-antimicrobial resistant organisms to humans, and calls for close monitoring in food animals and the environment to protect food animal consumers. It is also crucial for the stakeholders in the livestock industry to develop regulations and the efficient implementation of such standard guidelines or protocols to promote best farm practices and appropriate use of antimicrobials in Nigeria. Likewise, an undivided stakeholders’ collaboration (among the government, veterinary professionals, livestock farmers and LBS vendors) to tackle the issue of AMR and achieve the responsible use of AMU in the food animal production value chains (through a one health frame work) is much needed. The LBS’s two main preferred sources of information, which were radio and seminars, should be explored by local veterinary professionals and extension officers to promote antimicrobial stewardship for livestock owners. Strategies for efficient information dissemination and risk communication must be developed towards change in attitudes and behaviors among livestock farmers and vendors. Furthermore, the establishment of efficient surveillance systems for monitoring AMU and antimicrobial residues along the poultry production chain is very critical for reducing the emergence and risk of horizontal transmission of antimicrobial resistance to consumers and the environment. An increase in access to essential veterinary services must be enhanced and may be achieved through a commitment from the government towards capacity building and the training of more veterinarians and para veterinarians nationally. 

Several limitations were identified despite being the first to identify and describe risk factors associated with MDR in a live bird market-based setting in Abeokuta, Ogun State. These limitations restrict the external generalization of our findings. The sample size of respondents (LBS) was small, which may be due to the non- probabilistic convenience (voluntary) sampling approach. In addition, it took quite an effort to recruit the live bird vendors that participated in the study. Some of the vendors were enthusiastic about participating while others were skeptical due to the belief that the sample collection process could result in the death of the chickens. 

Our future work is to conduct a similar study at the state level and promote participation, representativeness of LBMs, and more conclusive quantitative data that will be generalizable. Similarly, it is important to conduct future studies especially genetic and nation-wide based surveillance, for a detailed understanding of the molecular epidemiology of MDR in live bird markets and the interconnected human population and the environment. 

## Figures and Tables

**Table 1:** Sociodemographic profiles of respondents (LBS) from the eight LBMs investigated in Abeokuta, Ogun State.

Variables	Frequency	Percent (%)	95% CI
Gender (*n* = 40)			
Male	6	15.0	6.7–29.5
Female	34	85.0	70.5–93.3
Marital Status (*n* = 39)			
Single	3	7.7	1.9–21.0
Married	36	92.3	79.0–98.1
Age (in years, *n* = 37)			
20–30	5	13.5	5.4–2.8
31–40	9	24.3	13.2–40.3
41–50	12	32.5	19.6–48.6
51–60	8	21.6	11.1–37.4
61–70	3	8.1	2.1–22.0
Educational level (*n* = 39)			
Informal	18	45.0	31.6–61.4
Primary	12	30.0	18.5–46.5
Secondary	9	22.5	12.4–38.5
Tertiary	1	2.5	<0.01–14.4
Primary Occupation [live bird, (*n* = 40)]			
No	3	7.5	1.9–20.6
Yes	92.5	37	79.4–98.1
Membership of the LBS Association (*n* = 40)			
No	5	12.5	5.0–26.6
Yes	35	87.5	73.4–95.0
Contact with a Vet (*n* = 39)			
Yes	23	59.0	43.4–72.9
No	16	41.0	27.1–56.6

**Table 2:** The distribution of responses to biosecurity and antimicrobial use question among LBS in Abeokuta, Ogun state (*n* = 40).

Questions/Variables	Options	Score	Responses (n)	(%)	95% CI
Biosecurity					
B1. Presence of wild chickens (*n* = 40)	Yes	0	21	52.5	37.5–67.1
	No	1	19	47.5	32.9–62.5
B2. Mix bird of different species (*n* = 40)	Yes	0	25	62.5	47.0–75.8
	No	1	15	37.5	24.2–53.0
B3. Mix chickens of different ages (*n* = 40)	Yes	0	19	47.5	32.93–62.5
	No	1	21	52.5	37.5–67.1
B4. Chickens are overcrowded (*n* = 40)	Yes	0	18	45	30.7–60.2
	No	1	22	55	39.8–69.3
B5. Cages are multi-layered (*n* = 40)	Yes	0	22	55	39.8–69.3
	No	1	18	45	30.7–60.2
B6. Inspection of chickens and processing facilities (*n* = 40)	Yes	1	8	20	10.2–35.0
	No	0	32	80	65.0–89.8
B7. Presence of sick chickens (*n* = 40)	Yes	0	17	42.5	28.50–57.8
	No	1	23	57.5	42.2–71.5
B8. Isolation pen for sick chickens (*n* = 40)	Yes	1	15	37.5	24.2–53.0
	No	0	25	62.5	47.0–75.8
B9. Introduce chickens without quarantine (*n* = 36)	Yes	0	31	77.5	70.9–94.4
	No	1	5	12.5	5.6–29.1
B10. Presence of other Animals (*n* = 40)	Yes	0	26	65	49.5–77.9
	No	1	14	35	22.1–50.6
B11. Source of Poultry (*n* = 39)	Various farms				
	Yes	0	25	62.5	49.5–77.9
	No	1	14	35	22.1–50.6
	Various LBS				
	Yes	0	22	55.5	41.0–70.7
	No	1	17	42.5	29.3–59.0
	Various Middlemen(n=39)				
	Yes	0	10	25.6	14.4–41.2
	No	1	29	74.4	58.8–85.6
B12. Cage Type (*n* = 40)	Metal	1	22	55	39.8–69.3
	Non-metal	0	18	45	30.7–60.2
B13. Clean the environment always (n = 40)	Yes	1	20	50	35.2 –64.8
	No	0	20	50	35.2–64.8
B14. Disinfect the environment always (*n* = 40)	Yes	1	16	40	26.3–55.4
	No	0	24	60	44.6–73.7
B15. Clean the cages always (*n* = 40)	Yes	1	19	47.5	32.9–62.5
	No	0	21	52.5	37.5–67.1
B16. Disinfect the cages always (*n* = 40)	Yes	1	10	25	14.0–40.4
	No	0	30	75	59.6–86.0
B17. Clean processing table regularly/always(*n* = 40)	Yes	1	17	42.5	28.5–57.8
	No	0	23	57.5	42.2–71.5
B18. Disinfect processing table regularly/always (*n* = 40)	Yes	1	8	20	10.2–35.0
	No	0	32	80	65.0–89.8
Antimicrobial use					
A1. Aware of antimicrobials (*n* = 40)	Yes	1	40	100	89.6–1.0
	No	0	0	0	0.0– 10.4
A2. Use Antimicrobials for poultry? (*n* = 40)	Yes	0	35	87.5	76.4–96.6
	No	1	5	12.5	3.4–23.6
A3. For what purpose? (*n* = 40)	Treat diseases				
	Yes	1	20	50	35.2–64.8
	No	0	20	50	35.2–64.8
	Prevent diseases				
	Yes	0	21	52.5	37.50–67.1
	No	1	19	47.5	32.9–62.5
A4. How do you administer drugs? (*n* = 40)	Call a veterinarian				
	Yes	1	4	10	3.4–23.6
	No	0	36	90	76.4–96.6
	Self				
	Yes	0	36	90	76.4–96.6
	No	1	4	10	3.4–23.6
A5. Where do you obtain antimicrobials for your chickens? (*n* = 40)	Vet Shops				
	Yes	1	22	55	39.8–69.3
	No	0	18	45	30.7–60.2
	Pharmacy shops				
	Yes	0	29	72.5	57.0–84.0
	No	1	11	27.5	16.0–43.0
	Poultry farmers				
	Yes	0	10	25	14.0–40.4
	No	1	30	75	59.6–86.0
	Live Bird Sellers				
	Yes	0	11	27.5	16.0–43.0
	No	1	29	72.5	57.0–84.0
A6. Are you influenced by company’s brand before use? (*n* = 40)	Yes	0	24	60	44.673.7
	No	1	16	40	26.3–55.4

**Table 3:** Sociodemographic information of LBS associated with biosecurity and AMU using bivariate analysis.

Variables	Biosecurity Level					AMU Level				
	Poor (%)	Satisfactory (%)	COR	95% CI	*p* Value	Poor (%)	Satisfactory (%)	COR	95% CI	*p* Value
Age (years) *n* = 37										
<46	17 (63.0)	2 (20.0)	6.8	1.20–38.56	0.03 ^b^*	14 (50.0)	5 (55.6)	0.8	0.17–3.61	1.00 ^b^
≥46	10 (37.0)	8 (80.0)				14 (50.0)	4 (4.4)			
Gender (*n* = 40)										
Male	6 (21.4)	0 (0.0)	-	-	-	6 (20.7)	0 (0.0)	-	-	-
Female	22 (78.6)	12 (100.0)				23 (79.3)	11 (100.0)			
Marital Status (*n* = 39)										
Single	2 (7.3)	1 (8.3)	0.88	0.07–10.75	1.00 ^b^	1 (3.6)	2 (18.2)	0.17	0.01–2.06	0.19 ^b^
Married	25 (92.6)	11 (91.7)				27 (96.4)	9 (81.8)			
Education (*n* = 40)								15.17	2.73–84.18	0.002 ^b^*
Informal/Primary	23 (82.1)	7 (58.3)	3.28	0.73–14.73	0.13 ^b^	26 (89.7)	4 (36.4)			
Post Primary	5 (17.9)	5 (41.7)				3 (10.3)	7 (63.6)			
Main Occupation as LBS (*n* = 40)								0.74	0.06–9.09	1.00 ^b^
Yes	28 (100.0)	9 (75.0)	-	-	-	27 (93.1)	10 (90.9)			
No	0 (0.0)	3 (25.0)				2 (6.9)	1 (9.1)			
Member of LBS association (*n* = 40)										
Yes	24 (85.7)	11 (91.7)	1.83	0.18–18.37	1.00 ^b^	24 (82.8)	11 (100.0)	-	-	-
No	4 (14.3)	1 (8.3)				5 (17.2)	0 (0.0)			

* Significant at *p* ≤ 0.05; ^b^ = Fischer’s exact test; COR, Crude’s odds ratio; CI, confidence interval.

**Table 4:** Distribution of *E. coli* and multidrug resistance.

LBM	*E. coli* Positive Samples (*n* = 32)	Prevalence of *E.coli* Isolates (%)	MDR *E. coli* Positive Samples (*n* = 18)	Prevalence of MDR *E. coli*(%)
Lafenwa	3	9.4	-	0.0
Kuto	4	12.5	2	11.1
Itoku	9	28.1	6	33.3
Gbonagun	1	3.1	1	5.6
Ago ika	3	9.4	2	11.1
Asero	4	12.5	2	11.1
Asejere	3	9.4	2	11.1
Osiele	5	15.6	3	16.6
Total	32		18	

**Table 5:** Multiple antimicrobial resistance indices (MARI) of antimicrobials used in live bird markets Abeokuta Ogun state.

S/N	Sample Code	Live Bird Market	Antimicrobial Class	MARI
1	KT 1AE(4)	Kuto	CL,2,3,4,5	0.5
2	ASEJ 3AE(4)	Asejere	CL,3,4,5,6	0.3
3	IT 7AE(4)	Itoku	CL,3,4,5,6	0.3
4	A1AE(3)	Ago Ika	CL,3,5,6	0.2
5	OS 3AE(3)	Osiele	CL,4,5,6	0.2
6	IT 6AE(3)	Itoku	CL,4,5,6	0.2
7	GB 2AE(4)	Gbonagun	CL,3,4,5,6	0.3
8	IT 8AE(3)	Itoku	CL,3,4,5	0.2
9	IT 3AE(3)	Itoku	CL,3,5,6	0.2
10	A 3AE(3)	Ago Ika	CL,3,4,5	0.2
11	IT 5AE(4)	Itoku	CL,3,4,5,6	0.3
12	OS 2AE(4)	Osiele	CL,3,4,5,6	0.3
13	AS 3AE(4)	Asero	CL,3,4,5,6	0.3
14	IT 4AE(4)	Itoku	CL,3,4,5,6	0.3
15	OS 5AE(4)	Osiele	CL,3,4,5,6	0.3
16	AS 1AE(3)	Asero	CL,4,5,6	0.2
17	KT 2AE(4)	Kuto	CL,3,4,5,6	0.2
18	ASEJ 2AE(4)	Asejere	CL,3,4,5,6	0.3

Class 1, Beta lactams cephalosporins (ceftriaxone, cefixime, cefuroxime, ceftazidime, cefotaxime); Class 2, fluoroquinolones (ciprofloxacin, ofloxacin, levofloxacin, nalidixic acid); Class 3, nitrofurans (nitrofurantoin); Class 4, beta lactamase inhibitor (amoxicillin clavulanate); Class 5, carbanapems (imipenem); Class 6, aminoglycosides (gentamicin). Classification was carried out based on the motor discs available for antimicrobial susceptibility testing.

**Table 6:** The Antimicrobial resistance among *E. coli* isolates from the eight LBMs in Abeokuta, Ogun State.

LBM	*E. coli* (*n* = 32)	NF (%)	CXM (%)	CRO (%)	ACXV (%)	ZEM (%)	LBC (%)	AUG (%)	CTX (%)	IMP (%)	OFX (%)	GN (%)	NA (%)	CAZ (%)	CPR (%)
Lafenwa	*n* = 3	33.3	0.0	0.0	0.0	0.0	0.0	0.0	0.0	33.3	0.0	0.0	33.3	66.7	0.0
Kuto	*n* = 4	50.0	50.0	0.0	75.0	0.0	50.0	50.0	0.0	100.0	50.0	25.0	100.0	100.0	75.0
Itoku	*n* = 9	77.7	44.4	11.1	55.5	0.0	22.2	66.6	11.1	100.0	0.0	55.5	44.4	100.0	33.3
Gbonagun	*n* = 1	100.0	100.0	100.0	100.0	0.0	0.0	100.0	0.0	100.0	0.0	100.0	100.0	100.0	100.0
Ago ika	*n* = 3	66.6	33.3	0.0	66.6	0.0	0.0	33.3	66.6	100.0	33.3	33.3	33.3	100.0	33.3
Asero	*n* = 4	50.0	25.0	0.0	25.0	100.0	0.0	75.0	0.0	75.0	0.0	75.0	75.0	100.0	50.0
Asejere	*n* = 3	100.0	100.0	33.3	66.6	33.3	0.0	66.6	33.3	100.0	0.0	66.6	33.3	100.0	33.3
Osiele	*n* = 5	40.0	100.0	0.0	80.0	20.0	0.0	60.0	60.0	100.0	0.0	80.0	20.0	100.0	20.0

ACX, Ampiclox; AUG, Amoxycillin/clavulanic acid; CAZ, Ceftazidime; CPR, Ciprofloxacin; CRO, Ceftriaxone; CTX, Cefotaxime; CXM, Cefuroxime; GN, Gentamicin; IMP, Imipenem; LBC-; NA, Nalidixic acid; NF, Nitrofurantoin; OFX, Ofloxacin; ZEM, Cefexime. Table S2. Antimicrobial profile of the positive *E. coli* isolates from live birds, Abeokuta Ogun State.

**Table 7:** Bivariate analysis of risk factors associated with MDR *E. coli* isolates collected from LBMs in Abeokuta, Ogun State.

Variables	MDR *E. coli*						
	Present	%	Absent	%	COR	95% CI	*p* Value
Use human AMs capsules for your birds							
Yes	15	78.9	12	80.0	0.94	0.17–5.02	0.64 ^b^
No	4	21.1	3	20.0			
AMU for treatment of sick poultry							
Yes	11	55.0	14	87.5	0.17	0.03–0.98	0.06 ^b^
No	9	45.0	1	12.5%			
AMU for prophylaxis							
Yes	13	65.0	12	81.3	8.04	1.69–38.13	0.01 ^a^*
No	7	35.0	3	18.7			
Veterinary Prescription							
Yes	2	10.0	1	6.3	1.66	0.137–20.23	1.00 ^b^
No	18	90.02	15	93.7			
Self-Prescription							
Yes	17	85.0	15	93.7%	0.38	0.03–4.03	1.00 ^b^
No	3	15.0	1	6.3			
Having contact with Vet							
Yes	14	63.6	9	52.9	1.56	0.43–5.64	0.50 ^a^
No	8	36.4	8	47.1			
Mix Poultry of various age							
Yes	8	35.4	11	61.1	0.36	0.10–1.32	0.12 ^a^
No	14	63.6	7	38.9			
Mix Poultry of various types							
Yes	14	63.6	11	61.1	1.11	0.31–4.03	1.00 ^a^
No	8	36.4	7	38.9			
Source poultry from various LBS							
Yes	12	57.1	10	55.6	1.07	0.30–3.79	1.00 ^a^
No	9	42.9	8	44.4			
Source poultry from various farms							
Yes	20	90.9	17	94.4	0.58	0.05–7.07	1.00 ^b^
No	2	9.1	1	5.6			
Presence of fence							
Yes	2	9.5	2	11.8	0.79	0.10–6.28	1.00 ^b^
No	19	90.5	15	88.2			
Presence of Wild birds							
Yes	9	40.9	8	44.4	1.16	0.33–4.07	0.82 ^a^
No	13	59.1	10	55.6			
Health Inspection							
Yes	4	18.2	4	22.2	0.79	0.16–3.67	1.00 ^b^
No	18	81.8	14	77.8			
Introduction of poultry without quarantine							
Yes	17	89.5	14	82.4%	1.82	0.26–12.47	0.65 ^b^
No	2	10.5	3	17.6			
Presence of sick poultry							
Yes	8	36.4	9	50.0	2.17	0.57–8.19	0.25 ^a^
No	14	63.6	9	50.0			
Cage Type							
Metal	10	45.5	12	66.7	0.42	0.12–1.51	0.18 ^a^
Non-metal	12	55.5	6	33.3			

* Significant at *p* ≤ 0.05; ^a^ = Pearson’s Chi Square; ^b^ = Fischer’s exact test; COR, Crude’s odds ratio; CI, confidence interval.

**Table 8:** The antimicrobials and concentrations of discs for susceptibility assay for *E. coli*.

Name of Antimicrobials	Class	Concentration Per Disc (μg)
Ceftriaxone	Cephalosporins	10
Cefixime		5
Cefuroxime		30
Ceftazidime		30
Cefotaxime		25
Nalidixic acid	Fluoroquinolones	30
Ciprofloxacin		5
Ofloxacin		5
Levofloxacin		5
Nitrofurantoin	Nitrofurans	300
Amoxicillin clavulanate	beta-lactam beta-lactamase inhibitor	30
Imipinem	Carbapenem	10
Gentamicin	Aminoglycosides	10

## Data Availability

Data are contained within the article or Supplementary Material.

## Supplementary Materials

The following are available online at https://www.mdpi.com/article/10.3390/antibiotics11020253/s1. Questionnaire S1: Questionnaire, Table S1: The Concentrations and Cut-Off Limits Used. For antimicrobials susceptibility testing, Table S2: Antimicrobial Profiles of the Positive *E. coli* Isolates from Live Chickens in Abeokuta Ogun State.
