# Supplementary material for: Multi-Drug Resistant Escherichia coli, Biosecurity and Anti-Microbial Use in Live Bird Markets, Abeokuta, Nigeria
Source: Antibiotics (Basel). 2022 Feb 16;11(2):253. doi: 10.3390/antibiotics11020253 (PMC8868421; doi:10.3390/antibiotics11020253)
Supplement: Supplementary file 1 [file antibiotics-11-00253-s001.zip › S1_Questionnaire.pdf]

FEDERAL UNIVERSITY OF AGRICULTURE ABEOKUTA
COLLEGE OF VETERINARY MEDICINE
DEPARTMENT OF PUBLIC HEALTH AND REPRODUCTION

LIVE BIRD SELLER QUESTIONAIRE

PROJECT TITLE: PREVALENCE AND PREDICTORS FOR MULTI-DRUG RESISTANT

Escherichia coli AMONG LIVE BIRDS IN ABEOKUTA, OGUN STATE

Date:

Introduction and consent section:

Dear Live Bird Seller,
I am Motunrayo Makanjuola, a final year veterinary student from the Federal University of Agriculture, Abeokuta.

What is the project about?
My research team is conducting a study that asks questions about working with chickens, what and how antimicrobials are used in the live bird market (LBM). We would also be collecting a little quantity of chicken faecal sample which will be tested for the presence of *E. coli* organism and the resistance to several antimicrobials. This bacterium is sometimes present in chickens, which are slaughtered at the LBM and the environment and of public health importance because it can be passed down to the meat and make consumers and sellers.

Why are we carrying out the project?
As you may be aware, the indiscriminate use of antimicrobials in food animals has been linked to the emergence of antimicrobial resistance in humans, which has become a worldwide health concern due to the associated increased morbidity and mortality in humans and animals.

Benefits of the project to farmers?
This study would provide baseline data for efficient development of educational programs and guidelines on AMU among live bird sellers, promote strategic planning or initiatives, and policy formulation or modifications where appropriate. All information gathered will be treated as highly confidential and will only be used for academic purposes.

Would you like to participate in this study? Yes [ ] No [ ]

Signature of respondent: ______________________
Date: _______________

Thank you for your time

1. GENERAL DATA ON THE LBM AND SAMPLE FLOCK

1. Date of the interview	
2. Name of Investigator	
3. Name of LBM	
4. Age of LBM	
5. Local Government Area	

6. GIS coordinates	
7. Types of Cages observed	Plastic crates [ ] Wooden cages [ ] Metal cages [ ] Rafia cages [ ] Others please specify ---
8. The investigator should record the average distance between cages	
9. Types of birds present	Layers [ ] Broilers [ ] Cockerels [ ] Guinea fowls [ ] Ducks [ ] Others [ ]<br>Please if others specify -------------------------------------
10. Presence of wild birds	Please mention --
11. Please write number of birds (flock size) present for each	Layers [ ] Broilers [ ] Cockerels [ ] Guinea fowls [ ] Ducks [ ] Others [ ]
12. Presence of various ages of birds	Yes [ ] No [ ]
13. Type of feeds	Locally compounded [ ] Commercial[ ]
14. Source of water	Borehole [ ] Well water [ ] Pipe borne [ ] others [ ] please specify-----------------------------------
15. Purpose for LBM	Please tick where appropriate<br>Selling [ ] Slaughtering [ ] Others [ ]<br>If others please specify ---
16. Are there any inspection and processing facilities for birds	Yes [ ] No [ ]
17. Presence of other Animals	Sheep [ ] Goat [ ] Cattle [ ] Fish [ ]<br>Others [ ] ---
18. Source of birds	Same farm[ ] Different farms [ ] LBMs [ ] others [ ]

2. POULTRY SELLER DEMOGRAPHICS

1. Age	Please specify------------------------
2. Gender	Male [ ] Female [ ]

3. Marital Status	Married [ ] Single [ ]
4. Education	Informal/Adult Education [ ] Primary[ ]<br>Secondary[ ] Tertiary [ ]
5. Is this your primary profession?	Yes [ ] No [ ]<br>If no, please specify occupation--------------------------------
6. Membership of poultry association	Yes [ ] No [ ]
7. Any contact with Veterinarian	Yes [ ] No [ ]
8. If yes, how frequent?	Everyday [ ] Once a week [ ]<br>Once a month [ ] others ……….
9. How long have you been a poultry seller?	Please specify-------------------------------------
10. How often do you clean the environment?	Always [ ] Very often [ ] Sometimes [ ]<br>rarely [ ] never [ ]
11. How often do you clean the bird cages?	Always [ ] Very often [ ] Sometimes [ ]<br>rarely [ ] never [ ]
12. How often do you clean table for chicken processing?	Always [ ] Very often [ ] Sometimes [ ]<br>rarely [ ] never [ ]
13. Explain briefly, procedures for cleaning?	
14. How do you dispose poultry Wastes?	Open dump sites [ ] Burial [ ] Burning [ ]<br>Nearby stream/river [ ] Others please specify --
15. Do you have separate cage for isolation of sick birds?	Yes [ ] No [ ]
16. You introduce birds into the market without quarantine	Yes [ ] No [ ]

17. Do you carry out emergency slaughter especially when birds are sick and sell?	Yes [ ] No [ ]
18. Do you wash your hands with soap after contact with poultry and their faeces?	All the time [ ] Sometimes [ ] Never [ ]
19. Do you wash your hands with soap after visiting the toilet?	All the time [ ] Sometimes [ ] Never [ ]

3. ANTIMICROBIAL USE AND RESISTANCE

1. Are you aware of Antibiotics/Antimicrobials?	
2. Do you use them for the birds?	Yes [ ] No [ ]
3. For what purpose?	Treatment [ ] Prevent diseases [ ] In feed [ ] In water [ ] others, please specify -------------------- ---
4. When do you treat birds?	When birds are sick [ ] when they are not sick [ ]
5. How do you treat birds?	Call a Vet [ ] Treat by yourself [ ] Live bird sellers' previous experience [ ] Others……….
6. Where do you obtain drugs for your birds?	Vet shops [ ] Pharmacy [ ] Poultry farmers [ ] Other live bird sellers [ ] Others [ ]
7. Are you influenced by company's brand before use?	Yes [ ] No [ ]

8. What influences your use of drugs?	Vet prescription [ ] Live bird sellers [ ] Previous experiences [ ] Sick birds [ ] Cost [ ] Others [ ]
9. Do you use human capsules for your birds?	Yes [ ] No [ ]
10. Do you observe withdrawal period for the antibiotics/antimicrobial before selling or slaughtering?	Yes [ ] No [ ]

11. S/N	Types	Yes	No	Rate of Use			Usage	
				Always	Occasionally	Never	Prophylactic	Therapeutic
1	Penicillin							
2	Gentamycin							
3	Enrofloxacin							
4	Streptomycin							
5	Tetracyclines							
6	Erythromycin							
7	Chloramphenicol							
8	Nalidixic							
9	Furazolidone							
10	Furataldone.							
11	Metronidazole							
12	Ampiclox							

12. Are you aware of antimicrobial resistance?	Yes [ ] No [ ] Not Sure [ ]
13. Are you aware of the implication in humans and animals?	Yes [ ] No [ ] Not Sure [ ]
14. Are you aware this could prolong treatment of sick birds and humans?	Yes [ ] No [ ] Not Sure [ ]

15. Are you aware this can lead to deaths in birds and humans?	Yes [ ] No [ ] Not Sure [ ]
16. Have you used antibiotics/antimicrobial in the last 2 days?	Yes [ ] No [ ]
17. Mention the antibiotics/antimicrobial used	
18. Have you slaughtered out of treated birds in the last 2 days	Yes [ ] No [ ]

4. PREFERRED INFORMATION CHANNEL ON ANTIMICROBIAL USE AND BIOSECURITY

**S/N**	**Channels/Sources**	**Yes**	**No**	**Appropriateness**		
				**Appropriate**	**Inappropriate**	**I don't know**
1	Radio					
2	Television					
3	Internet					
4	Seminar/Workshop					
5	Extension Officer					
6	Veterinary Officer					
7	Media platforms e.g. Whatsapp, twitter, facebook					
8	Colleagues/Friends					
9	Research Institute					
10	Others					
