# Supplementary material for: Multi-Drug Resistant Escherichia coli, Biosecurity and Anti-Microbial Use in Live Bird Markets, Abeokuta, Nigeria
Source: Antibiotics (Basel). 2022 Feb 16;11(2):253. doi: 10.3390/antibiotics11020253 (PMC8868421; doi:10.3390/antibiotics11020253)
Supplement: Supplementary file 1 [file antibiotics-11-00253-s001.zip › Table S1 and Table S2.docx.pdf]

Table S1.

The Concentrations and Cut-Off Limits Used For Antimicrobials Susceptibility Testing.

Name of antimicrobials	Class	Concentration per disc (μg)	S	I	R
Ceftriaxone	Cephalosporins	10	≥19	20-22	≤23
Cefixime		5	≥19	16-18	≤15
Cefuroxime		30	≥23	15-22	≤14
Ceftazidime		30	≥18	15-17	≤14
Cefotaxime		25	≥19	20-22	≤23
Nalidixic acid	Fluoroquinolones	30	≥19	14-18	≤13
Ciprofloxacin		5	≥21	16-20	≤15
Ofloxacin		5	≥16	13-15	≤12
Levofloxacin		5	≥17	14-16	≤13
Nitrofurantoin	Nitrofurans	300	≥17	15-16	≤14
Ampiclox	Beta-lactamase	30	≤13	14-16	≥17
Amoxicillin clavulanate		30	≤13	14-17	≥18
Imipinem	Carbapenem	10	≥23	20-22	≤19
Gentamicin	Aminoglycosides	10	≥15	13-14	≤12

Table S2

Antimicrobial Profile of the Positive *E. coli* Isolates from Live Birds Abeokuta Ogun State.

	SAMPLE ID	MDR	NF	CXM	CRO	ACX	ZEM	LBC	AUG	CTX	IMP	OFX	GN	NA	CAZ	CPR
1	KT 1AE	+VE	R	R	S	R	S	R	R	S	R	R	S	R	R	R
2	KT 4AE	- VE	I	R	S	R	S	R	S	S	R	R	S	R	R	R
3	ASEJ 3AE	+VE	R	R	R	R	S	S	R	S	R	S	R	I	R	R
4	IT 7AE	+VE	R	R	S	R	S	S	R	R	R	S	R	R	R	R
5	A1AE	+VE	R	I	S	R	S	I	I	R	R	R	R	R	R	R
6	OS1AE	-VE	I	R	S	R	S	S	I	S	R	S	S	S	R	S
7	OS3AE	+VE	I	R	S	R	S	S	R	S	R	S	R	S	R	S
8	IT 6AE	+VE	I	I	S	R	S	S	R	S	R	S	R	S	R	S
9	IT2AE	-VE	S	I	S	S	S	R	R	S	R	S	S	I	R	R
10	GB2AE	+VE	R	R	R	R	S	S	R	I	R	S	R	R	R	R
11	IT 8AE	+VE	R	I	S	R	S	S	R	S	R	S	S	S	R	S
12	A2AE	-VE	S	I	S	S	S	S	I	S	R	S	S	S	R	S
13	IT3AE	+VE	R	R	S	R	I	S	S	S	R	S	R	R	R	S
14	IT9AE	-VE	R	S	S	R	S	S	S	S	R	S	S	R	R	S
15	A3AE	+VE	R	R	S	R	S	S	R	R	R	S	S	S	R	S
16	IT 5AE	+VE	R	R	S	S	S	S	R	S	R	S	R	I	R	S
17	OS2AE	+VE	R	R	S	S	S	S	R	R	R	S	R	S	R	R
18	AS3AE	+VE	R	R	S	S	S	I	R	S	R	S	R	R	R	R
19	AS 4AE	-VE	S	S	S	S	S	S	S	S	R	S	R	R	R	R
20	IT4AE	+VE	R	R	R	S	S	S	R	S	R	S	R	R	R	R
21	OS6AE	-VE	S	R	S	R	R	S	I	R	R	S	R	I	R	S
22	L 1AE	-VE	S	S	S	S	S	S	S	S	S	S	S	S	S	S
23	IT1AE	-VE	R	S	S	S	S	R	S	S	R	S	S	S	R	S
24	OS 5AE	+VE	R	R	S	R	S	S	R	R	R	S	R	R	R	I
25	AS1AE	+VE	S	S	S	R	S	S	R	S	R	S	R	S	R	S
26	L3AE	-VE	S	I	S	S	S	I	S	S	R	S	S	R	R	S
27	KT2AE	+VE	R	R	S	R	S	S	R	S	R	I	R	R	R	R
28	L 2AE	-VE	R	S	S	S	S	S	I	I	S	S	S	S	R	S

	SAMPLE ID	MDR	NF	CXM	CRO	ACX	ZEM	LBC	AUG	CTX	IMP	OFX	GN	NA	CAZ	CPR
29	AS2AE	-VE	R	S	S	S	S	S	R	I	I	S	S	R	R	I
30	ASEJ 1AE	-VE	R	R	S	S	S	S	I	R	R	S	S	S	R	S
31	ASEJ 2AE	+VE	R	R	S	R	R	S	R	R	R	S	R	R	R	I
32	KT3AE	-VE+	S	S	S	S	S	S	S	S	R	S	S	R	R	I
